# Silent intrathyroid parathyroid carcinoma

**DOI:** 10.1530/EDM-23-0027

**Published:** 2023-05-15

**Authors:** Ekaterina Kim, Ekaterina Bondarenko, Anna Eremkina, Petr Nikiforovich, Natalia Mokrysheva

**Affiliations:** 1 Endocrinology Research Centre, Moscow, Russia

**Keywords:** Adult, Male, White, Russian Federation, Parathyroid, Endocrine-related cancer, Unique/unexpected symptoms or presentations of a disease, May, 2023

## Abstract

**Summary:**

A 59-year-old male presented with an accidental thyroid mass in 2022. Ultrasound and CT scan showed a nodule 5.2 × 4.9 × 2.8 cm (EU-TIRADS 4) in the right lobe of the thyroid gland. Taking into account the results of the fine needle aspiration biopsy (Bethesda V), intrathyroid localization, and absence of clinical symptoms, a malignant tumor of the thyroid gland was suspected. The patient underwent total thyroidectomy using fluorescence angiography with indocyanine green, and two pairs of intact parathyroid glands were visualized in typical localization. Unexpected histological and immunohistochemistry examinations revealed parathyroid carcinoma. Due to the asymptomatic course of the disease and atypical localization of parathyroid tumor, primary hyperparathyroidism was not suspected before the surgery. The diagnosis of asymptomatic intrathyroid parathyroid cancer is a serious diagnostic challenge for a wide range of specialists.

**Learning points:**

- Parathyroid cancer is a rare disease that may be asymptomatic.
- Intrathyroidal localization of parathyroid carcinoma is casuistic and challenging for diagnosis, and the treatment strategy is not well defined.
- Preoperative parathyroid hormone and serum calcium testing are recommended for patients with solid thyroid nodules (Bethesda IV–V).

## Background

Parathyroid carcinoma (PC) is a rare malignant, usually hormonally active neoplasm which accounts for <1% of all the cases of primary hyperparathyroidism (PHPT) (1) characterized by excessive secretion of parathyroid hormone (PTH) and hypercalcemia. However, rare cases of non-functioning PC have been described. Recently, the prevalence of asymptomatic PHPT has risen progressively to 10–24% (2) possibly related to improved laboratory screening. The pathogenesis of PC remains unclear. PC is more often sporadic, and less often gene mutations are detected. According to the literature, ectopic glands can occur in up to 22% of cases. One of the most common ectopic locations of the parathyroid glands (PTGs) is thyroid tissue (33.0%) (3) because of closely related embryogenesis of the PTGs and thyroid glands. Preoperative diagnosis of PC, especially in the case of intrathyroidal location, entails a number of diagnostic difficulties. Fine needle aspiration biopsy (FNAB) is the most informative study of thyroid nodules; however, cytological features of parathyroid and thyroid lesions are similar which can lead to misinterpretation (4). The diagnosis of PC can be established only by the results of histological examination. A combination of intrathyroid parathyroid cancer and asymptomatic course leads to a difficult diagnostic search, which can affect treatment strategy and outcomes.

## Case presentation

**Figure 1:**
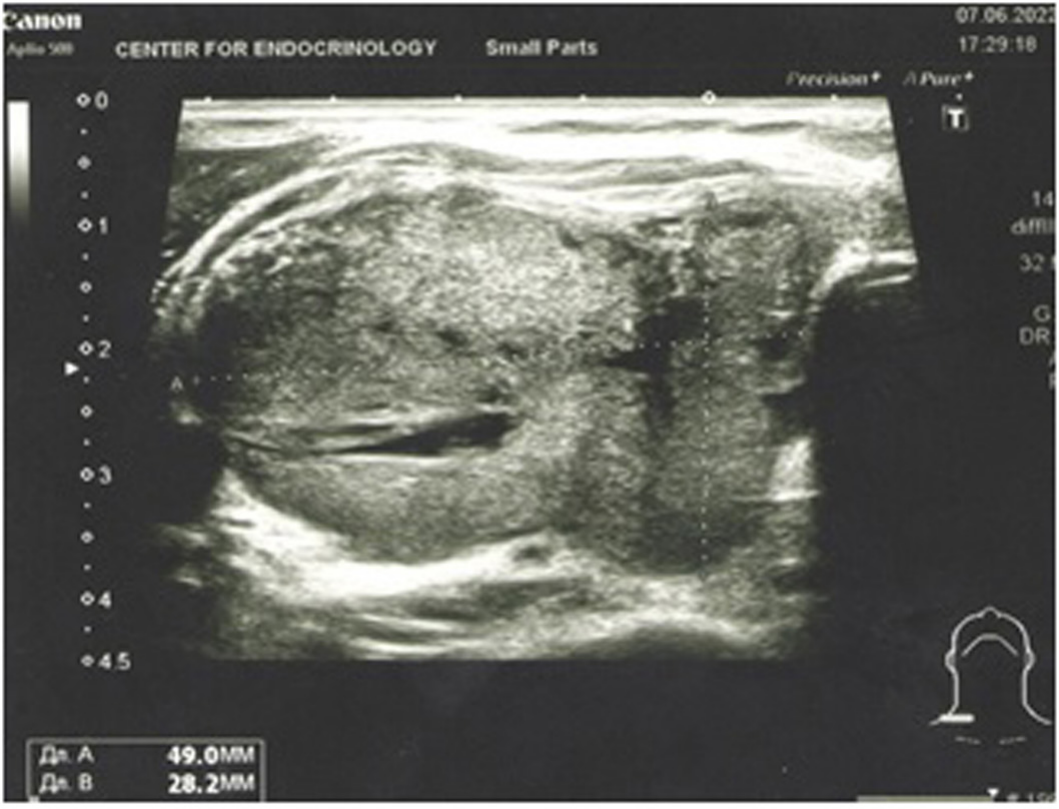
US of thyroid gland showing solid mass 5.2 × 4.9 x× 2.8 cm in the right lobe.

We present the case of a 59-year-old male initially complaining about feeling a ‘lump in the throat’. A thyroid nodule had incidentally been detected during a chest CT scan in April 2022. Subsequently, thyroid ultrasonography (US) showed hypoechoic mass 5.2 x× 4.9 × 2.8 cm with irregular contours, anechoic zones, and calcifications (EU-TIRADS 4) in the right lobe; isoechoic nodule in the left lobe 0.7 cm (EU-TIRADS 3); and tracheal displacement to the left (Fig. 1).

## Investigation

US was followed by FNAB of the node in the right thyroid lobe which revealed numerous groups of neoplasm cells formed papillary structures that do not allow to exclude the papillary carcinoma (Bethesda V). According to a laboratory study: calcitonin < 2 pg/mL, TSH – 0.6 mIU/L (0.4–4.0), T4 – 13.23 pmol/L (9–19), T3 – 3.93 pmol/L (2.6–5.7); PTH and serum calcium levels were not measured.

## Treatment

**Figure 2:**
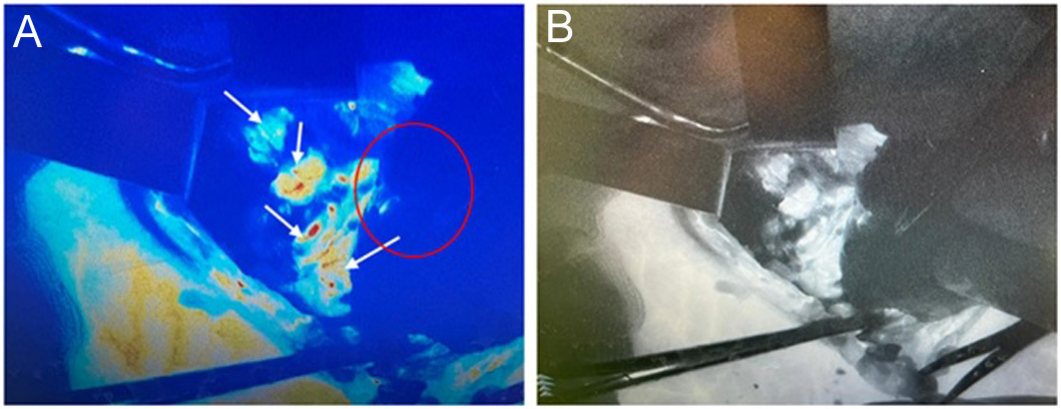
Intraoperative ICG navigation. (A) intensive blood supply to the parathyroid glands in a typical location in the color spectrum (→), no accumulation in the tumor (red round), 90 s after intravenous administration of ICG; (B) the infrared spectrum with the light off.

Subsequently, given the size of the thyroid nodule and FNAB results, our patient underwent a total thyroidectomy with an intraoperative navigation in July 2022. Angiography with the indocyanine green (ICG) was performed. Typical ICG enrichment in PTGs allowed us to detect two pairs of intact PTGs in a typical location (two PTGs a the right side and two at the left side) (Fig. 2). Additionally, there was the intrathyroid ICG-negative high-density tumor 5.0 × 4.0 × 5.0 cm located at the anterior surface of the right thyroid lobe.

**Figure 3:**
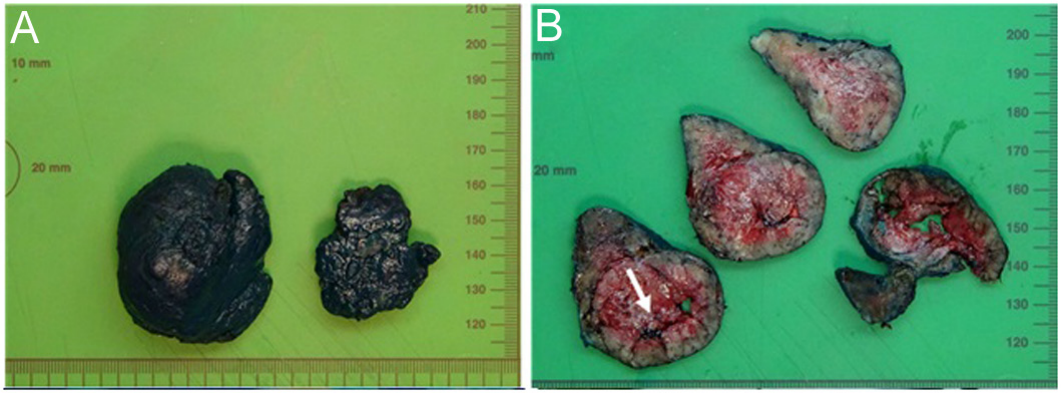
Macroscopic images. (A) Significant increase in the right lobe of the thyroid gland compared to the left; (B) the right lobe is represented by the formation of a pink-brown lobed structure with extensive hemorrhage in the center (→).

**Figure 4:**
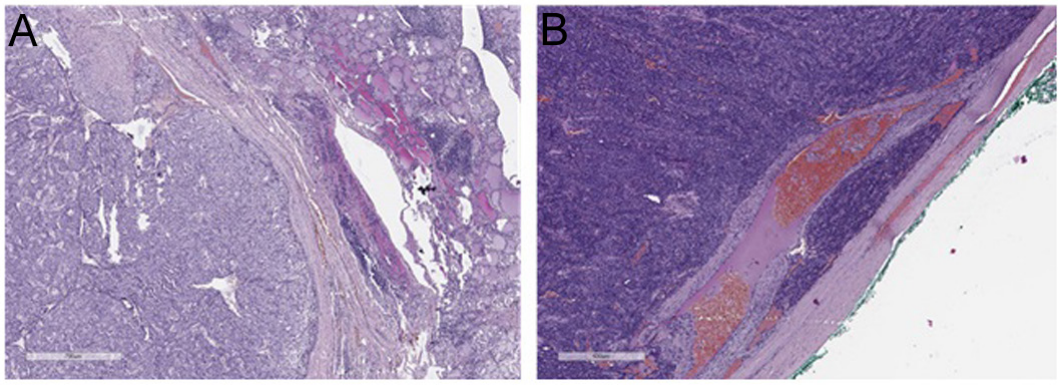
Histopathology (H&E). (A) A tumor of a solid trabecular structure covered by a fibrous capsule; surrounding thyroid tissue with lymphocytic infiltration; (B) vascular invasion in the capsule.

**Figure 5:**
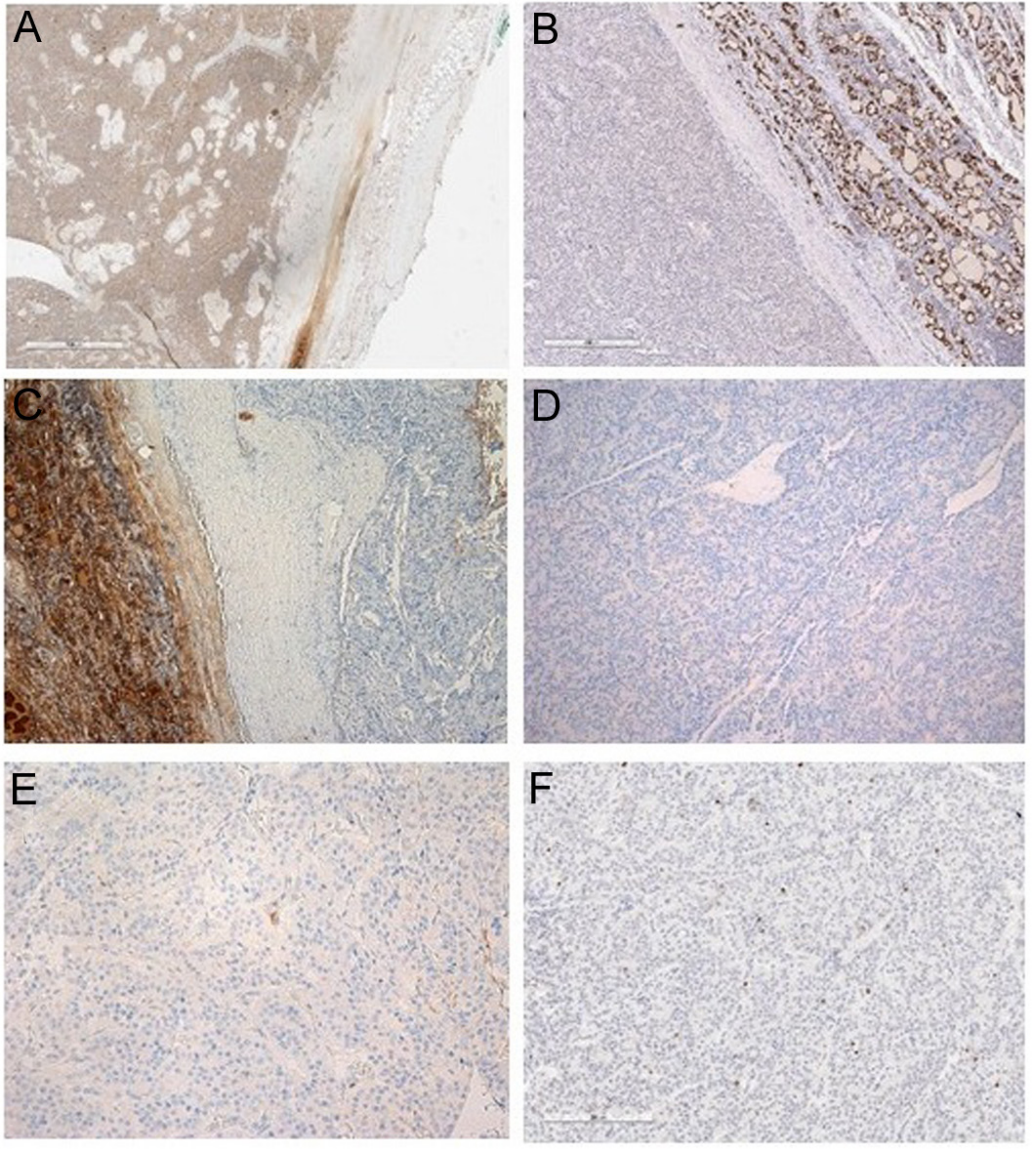
IHC examination. (А) Diffuse expression of PTH in the tumor, negative in the surrounding tissue; negative expression of TTF-1 (B) and thyroglobulin (С) in the tumor; positive expression of TTF-1 (B) and thyroglobulin (C) in the surrounding thyroid tissue; negative CK7 (D) and HBME-1 (E) expression in the tumor; (F) Ki-67 5%.

Histopathological evaluation showed an irregular gray nodule in the right thyroid lobe with central hemorrhage (Fig. 3) about 4.0 cm in size, consisting of cells with hyperchromatic nuclei. Besides, it had multiple foci of vascular invasion (Fig. 4A and B). Surrounding thyroid tissue of micro-normo-follicular architecture had lymphocytic infiltration without signs of tumor spread (Fig. 4A). The tumor was positive for PTH (Fig. 5A) and negative for TTF-1 (Fig. 5B), thyroglobulin (Fig. 5C), CK7 (Fig. 5D), and HBME-1 (Fig. 5E) confirming that it was indeed a parathyroid neoplasm. Ki-67 was up to 5% (Fig. 5F). The presence of reliable signs of vascular and capsular invasion and the results of the IHC study allowed us to diagnose PC pT1 (AJCC 8th).

## Outcome and follow-up

Severe hypocalcemia developed in the postoperative period: calcium ionized 0.97 mmol/L (1.03–1.29), calcium total 2.02 mmol/L (2.15–2.55), and PTH 1.91–6.07 pg/mL (15–65). The patient complained about severe numbness in the arms and legs. Alfacalcidol 1 mcg, calcium carbonate 1 g, and levothyroxine sodium 175 mcg per day were prescribed with a positive effect.

Then, we performed a genetic study to clarify the etiology of PC. Gene panel sequencing including 27 genes associated with hereditary forms of PHPT (*AIP, AP2S1, CASR, CDC73, CDKN1A, CDKN1B, CDKN1C, CDKN2A, CDKN2C, CDKN2D, DICER1, FAM111A, GATA3, GCM2, GNA11, GNAS, MEN1, POU1F1, PRKAR1A, PRKCA, PTEN, PTTG2, SDHA, SDHB, SDHC, SDHD,* and *TBCE*) did not reveal any mutations.

Nine months after surgery, the patient remains in biochemical remission of the disease (calcium ionized: 1/26 mmol/L (1.18–1.32), calcium total: 2.42 mmol/L (2.15–2.5), and PTH: 1.14 pmol/L (1.7–6.4)). He continued to take alfacalcidol 1 mcg/day, calcium carbonate 2,000 mg/day, and cholecalciferol 10 000 IU/week to maintain normal calcium phosphorus metabolism.

## Discussion

Diagnosis of intrathyroid PC is challenging, especially in the case of asymptomatic PHPT (1). Usually, there are two pairs of PTGs, but the incidence of supernumerary glands occurs on average in 15% of cases (range 3–25%), wherein their number can reach 12 pieces. Intrathyroidal location of PC is extremely rare, about 20 such cases have been described in the literature (5, 6). Intrathyroid PC was more common in women (*n* = 14) with a median age of diagnosis of 56 (36; 63) years. We did not find a single case with tumor infiltration of the affected supernumerary PTG. In most of the described cases of intrathyroid PC, patients presented with symptoms associated with hyperparathyroidism and hypercalcemia. Asymptomatic PC represents no more than 2% of all cases (5, 7), while usually manifestation related to local growth and enlarged thyroid gland.

Benali *et al.* presented a female patient with thyroid goiter who underwent total thyroidectomy without lymph node dissection. Pathological examination revealed PC with positive resection margins. Imaging methods showed residual tissue; however, the patient refused further surgery and therefore was exposed to radiation with the remission achievement. The case demonstrates initial misdiagnosis of PC contributed to further therapeutic difficulties (5). The success of surgery determines the patient's prognosis. In the case of secondary foci, surgical removal remains the only effective method due to their low sensitivity to cytotoxic chemo- and radiotherapy.

In the present case, the thyroid nodule was initially assumed on chest CT. The subsequent US also supported the initial diagnosis because the echographic features of intrathyroid parathyroid tumors and thyroid nodules are similar. Moreover, the main method for stratifying thyroid lesions remains FNAB which does not allow to distinguish parathyroid and thyroid tumors due to the morphological similarity (presence of papillary-like architectures, microfollicular cells, macrophages, colloid-like material, oxyphilic cytoplasm, and naked nuclei) (4). Therefore, the results of FNAB in our case were regarded as papillary thyroid cancer.

ICG angiography is applied for the intraoperative identification of the PTG during thyroidectomy. PTGs tend to accumulate ICG, but are not targeted. The intrathyroid location of the PTG hinders ICG detection due to interference from background thyroid fluorescence that leads to false-negative results of intraoperative monitoring. Also, few studies showed that benign PTG had a higher target-to-background ratio compared to malignancy neoplasms. There is a trend toward a higher ICG fluorescence in patients with preoperative PTH levels > 1900 pg/mL (*P* < 0.05) and PTGs larger than 10 mm (*P* < 0.01) (8).

Given the asymptomatic course, the absence of hypercalcemia symptoms, serum calcium, and PTH was not tested at the preoperative stage. Also, two pairs of PTGs were visualized in typical places during the surgery, so we did not exam PTH and serum calcium intraoperatively.

Poortmans *et al.* described a young woman with PC who was asymptomatic despite severe hypercalcemia. Nevertheless, preoperative calcium and PTH measurements allowed to suspect a parathyroid tumor. Further intraoperative laboratory monitoring was used to evaluate the effectiveness of surgery (5). In our case, severe hypocalcemia after surgery with the remaining intact PTGs indicated hormonal activity of PC. Probably the remaining glands were suppressed by hormonally active PC.

The diagnosis of PC is based on histological examination; however, in some cases, additional IHC analysis can be used. According to the 2022 WHO classification of endocrine and neuroendocrine tumors, the main diagnostic criteria of PC are still reliable signs of invasion: vascular and/or lymphatic and/or perineural and/or neighboring structures/organs, as well as the presence of confirmed metastases (9). In the presented case, there was no doubt about the malignant nature of the tumor; however, the positive expression of PTH and the absence of TTF-1, thyroglobulin, CK7, and HBME-1 expression made it possible to clarify its origin.

PC is typically sporadic but also can be part of hereditary syndromes (10). We provided genetic testing to exclude family forms; however, no mutations associated with PHPT were identified.

The 10-year survival rate for PC is about 50–70% (10). The frequency of tumor recurrence after surgical treatment is approximately 40–60%. PC more often affects nearby tissues, distant metastases are less common. Considering the volume of primary surgery, negative resection margins, and biochemical remission, the patient's prognosis is favorable. We recommended regular follow-up of calcium–phosphorus metabolism.

This clinical case is of particular interest to both surgeons and endocrinologists determining the patient management tactics. The absence of reliable preoperative diagnostic criteria, especially in the case of intrathyroidal localization, makes the diagnosis of PC extremely difficult. Correct preoperative examination is crucial since en bloc surgery is the only potentially curative therapy for PC.

In conclusion, thyroid nodules should cause suspicion regarding the presence of an intrathyroidal parathyroid lesion. Due to the low information value of US and FNAB for differential diagnosis between thyroid and intrathyroidal PC, it is preferable to evaluate serum calcium levels in all patients with a nodular goiter planning surgical treatment. Using needle-washing liquid, PTH measurement can increase the information content of FNAB in cases of dispute.

## Declaration of interest

The authors declare that there is no conflict of interest that could be perceived as prejudicing the impartiality of the research reported.

## Funding

This research was funded by the state assignment № АААА-А18 -121030100032-7.

## Patient consent

Written informed consent for publication of the clinical details and clinical images was obtained from the patient.

## Author contribution statement

E Kim was involved in the literature review and writing of the paper; E Bondarenko examined histopathological specimens, delivered a definitive diagnosis, and provided a paper review; A Eremkina provided a paper review; P Nikiforovich performed the surgery and captioned the intraoperative images included in the manuscript; N. Mokrysheva was involved in the final paper review.
